# Research on improved algorithm for helmet detection based on YOLOv5

**DOI:** 10.1038/s41598-023-45383-x

**Published:** 2023-10-23

**Authors:** Chun Shan, HongMing Liu, Yu Yu

**Affiliations:** 1 Guangdong Polytechnic Normal University, Guangzhou, China; 2 Guangzhou University, Guangzhou, China

**Keywords:** Computational biology and bioinformatics, Energy science and technology, Engineering, Mathematics and computing

## Abstract

The continuous development of smart industrial parks has imposed increasingly stringent requirements on safety helmet detection in environments such as factories, construction sites, rail transit, and fire protection. Current models often suffer from issues like false alarms or missed detections, especially when dealing with small and densely packed targets. This study aims to enhance the YOLOv5 target detection method to provide real-time alerts for individuals not wearing safety helmets in complex scenarios. Our approach involves incorporating the ECA channel attention mechanism into the YOLOv5 backbone network, allowing for efficient feature extraction while reducing computational load. We adopt a weighted bi-directional feature pyramid network structure (BiFPN) to facilitate effective feature fusion and cross-scale information transmission. Additionally, the introduction of a decoupling head in YOLOv5 improves detection performance and convergence rate. The experimental results demonstrate a substantial improvement in the YOLOv5 model's performance. The enhanced YOLOv5 model achieved an average accuracy of 95.9% on a custom-made helmet dataset, a 3.0 percentage point increase compared to the original YOLOv5 model. This study holds significant implications for enhancing the accuracy and robustness of helmet-wearing detection in various settings.

## Introduction

Intelligent upgrading and management of traditional industrial parks represent a new model for industrial development aimed at promoting industrial transformation, enhancing productivity, improving resource utilization efficiency, and ensuring environmental sustainability. While wearing a helmet is crucial to protect workers' heads from falling objects and potential collisions on complex construction sites, it is often observed that construction workers lack safety awareness and do not consistently wear helmets for various reasons. This behavior not only poses a significant threat to workers' personal safety but also hinders effective supervision at construction sites. Therefore, there is an urgent need to find more effective methods to monitor and enforce helmet usage.

Traditional supervision methods predominantly rely on manual oversight, which consumes substantial material and human resources. In recent years, many scholars, both domestically and internationally, have applied machine learning technology to helmet detection. This approach can significantly reduce labor costs while ensuring effective supervision. For instance, Lowe^1^ proposed a scale-invariant feature transformation method for detecting key image features. Viola et al.^2^ developed the Haar-like feature extraction method by exploring human facial features . To enhance feature extraction efficiency, directional gradient histograms have also been employed for target detection^3^. In 2014, R. Girshick and colleagues introduced the Region-Convolutional Neural Network (RCNN) algorithm, marking the acceleration of target detection development with convolutional neural networks^4^.

Due to human limitations in processing large amounts of loosely correlated information simultaneously, researchers have translated this concept into deep learning to extract more useful features and enhance target detection model performance. This mechanism is known as the attention mechanism and is widely used in various fields, such as natural language processing^5^, computer vision, and tasks like face recognition, crowd detection, car recognition, insulator detection, and speech recognition^6–10^. In 2017, Hu et al. introduced the Squeeze and Excitation Network (SENet)^11^. Subsequently, in 2018, Park et al. proposed the Convolutional Block Attention Module (CBAM) and Bottleneck Attention Module (BAM), each featuring channel and spatial attention modules^12,13^. These modules have been integrated into the ResNet network, providing plug-and-play convenience to improve feature quality.

Our research will delve into the principles and methods of a fusion attention mechanism, as well as enhance the YOLO algorithm across multiple stages and levels. This enhancement aims to enable the model to better extract features and process complex data, ultimately improving its accuracy, stability, and generalization capabilities. To validate the model's effectiveness, we will conduct experiments and compare its performance with other attention models. Finally, we will discuss potential challenges and future directions for this improved model. We hope to offer an efficient and accurate helmet detection solution for industrial, construction, and manufacturing fields, thereby contributing to the safeguarding of employees' personal safety and the reduction of workplace accidents. Simultaneously, we anticipate that this paper will inspire further exploration of target detection technology applications in safety-related fields, fostering innovation and development. The main contributions of this paper are summarized as follows:

1. Due to the lack of a large number of public safety helmet detection datasets in field scenarios, an open-source safety helmet dataset was created using Mosaic data augmentation.
2. Considering the high computational load and memory requirements of YOLOv5, making it challenging to deploy on small mobile embedded devices, improvements were made to the backbone network, specifically the BIFPN structure. Additionally, the Efficient Channel Attention was introduced. Comparative experiments were conducted using the dataset at each improvement point to validate their effectiveness.
3. In response to the time-consuming and cumbersome safety helmet detection process in existing research, this paper introduced the YOLOX decoupled head structure. This structure separates the extraction of target position and category information, enabling them to be learned independently in different network branches. This approach effectively reduces the number of parameters and computational complexity, greatly enhancing convergence speed, model generalization, and robustness.

The rest of this paper is organized as follows.: “Related work” provides a review of related work. In “Improved methods”, we elucidate the proposed improved methods. “Experiment and results” delves into experiments and results, with conclusions drawn in “Conclusion”.

## Related work

In addition to the methods outlined above, numerous studies have dedicated their efforts to harnessing deep learning networks for the extraction of highly discriminative features in helmet detection^14^. These studies underscore the integration of diverse data sources and the utilization of knowledge graphs for comprehensive data analysis and inference, ultimately leading to a more profound comprehension of the intricate interrelationships within the data^15^. Furthermore, the focal point of future research is expected to shift towards cybersecurity concerns and the deployment of edge devices in industrial IoT systems^16^. Notably, Sun et al.^17^ have introduced a method for honeypot identification based on deep learning, while Tian et al.^18^ have unveiled a digital evidence framework founded on blockchain technology. Both of these approaches hold pivotal roles in ensuring the security, immutability, and traceability of the digital evidence subject to examination.

Deep learning-based target detection algorithms can be primarily categorized into one-stage detection algorithms and two-stage detection algorithms. Within the one-stage category, renowned options include the YOLO (You Only Look Once) series^19^ and SSD (Single Shot MultiBox Detector)^20^. These algorithms excel in their ability to efficiently extract features and employ multi-scale detection techniques, thereby facilitating real-time target detection with a high degree of accuracy. In contrast, two-stage detection algorithms involve a two-step process, first localizing the object through a candidate box, followed by classifying the localized content. Representative examples encompass RCNN, SPP-NET, Fast R-CNN, Faster R-CNN, Mask R-CNN, and Cascade R-CNN^21–26^. While two-stage detection algorithms offer exceptional accuracy, their real-time performance often falls short, rendering one-stage detection algorithms the prevailing choice. Ongoing research endeavors have given rise to numerous one-stage detection algorithms, each endowed with distinctive features that enhance detection accuracy without compromising processing speed. YOLOv5, as the most recent optimization within the YOLO series, has garnered significant attention for its exceptional performance and efficient real-time processing. It achieves higher accuracy and faster speeds, rendering it an ideal solution for the helmet detection challenge.

The YOLO series algorithm framework^27^, a classic one-stage target detection algorithm, conducts detection within a single stage, simplifying the operational process and enhancing speed. YOLOv5 introduces four network models: YOLOv5s, YOLOv5m, YOLOv5l, and YOLOv5x. Among these, YOLOv5s features the smallest network volume, whereas YOLOv5m, YOLOv5l, and YOLOv5 increase the depth and width of the network structure, resulting in improved accuracy, albeit with a slightly slower processing speed.

The YOLOv5 algorithm model is divided into Input, Backbone, Neck, Head, and Output, typically based on the CSP architecture^28^. The Backbone network extracts features from input images with fewer parameters, enabling faster inference speed. Additionally, YOLOv5 incorporates a feature pyramid network after the Backbone network to handle targets of various scales. This network uses multiple feature maps of different levels to detect targets of different sizes. YOLOv5 generates candidate boxes by applying anchor boxes to represent targets of varying sizes and aspect ratios. Each feature layer includes a prediction layer, which outputs target confidence, class probability, and box location information for class and location prediction. Training the YOLOv5 model requires a loss function to measure the difference between predicted results and true labels, with YOLOv5 using a comprehensive loss function that includes confidence loss, class loss, and box regression loss. In the final object detection stage, non-maximum suppression (NMS) removes overlapping detection boxes and selects the detection result with the highest confidence as the final output.

To validate the effectiveness of the improved model and the proposed YOLOv5 target detection algorithm, we conducted comparative experiments on the dataset. Considering practical application scenarios, this paper focuses on integrating all improvements into an overall model and validating it using real-world scenario datasets. We aim to assess the improved algorithm's detection performance under actual conditions. The next section will introduce the process of improving the original algorithm.

## Improved methods

To enhance the applicability of the YOLOv5 algorithm for helmet detection in complex environments, several optimizations are needed. Firstly, adopting a lightweight backbone network for feature extraction is essential before deploying the algorithm on the target device. Simultaneously, by incorporating the improved Neck network to extract more intricate features, we can enhance the algorithm's ability to focus on small targets, expedite convergence, and predict outcomes efficiently through the Head computation. We achieve this by integrating the ECA channel attention mechanism, which efficiently extracts features from the input image using fewer parameters, resulting in faster inference speeds.

Additionally, to address targets of varying scales, we retain the feature pyramid network^29^, as in the original YOLOv5. This network utilizes multiple feature maps from different layers to detect targets of different sizes. To enrich the feature mapping with semantic information, we have adopted the BiFPN feature fusion structure within the Neck section of the network, replacing the FPN + PAN feature fusion structure from the original YOLOv5 target detection algorithm.

**Figure 1:**
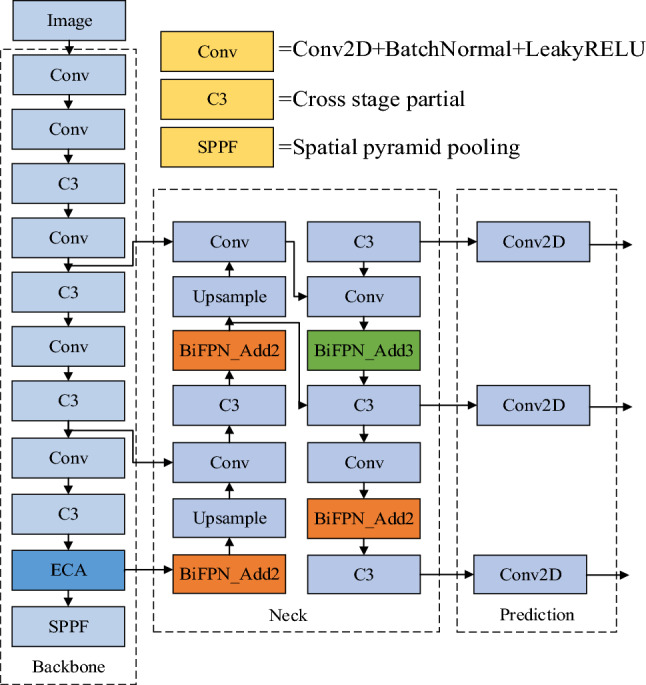
Improved YOLOv5 network architecture diagram.

For generating candidate boxes representing targets of different sizes and aspect ratios, YOLOv5 continues to utilize anchor boxes. Furthermore, to alleviate conflicts between the classification and localization branches in the target detection task, we transitioned from a shared header structure to a decoupled header. You can find a visual representation of the improved YOLOv5 network structure in Fig. 1.

### A. Data enhancement

**Figure 2:**
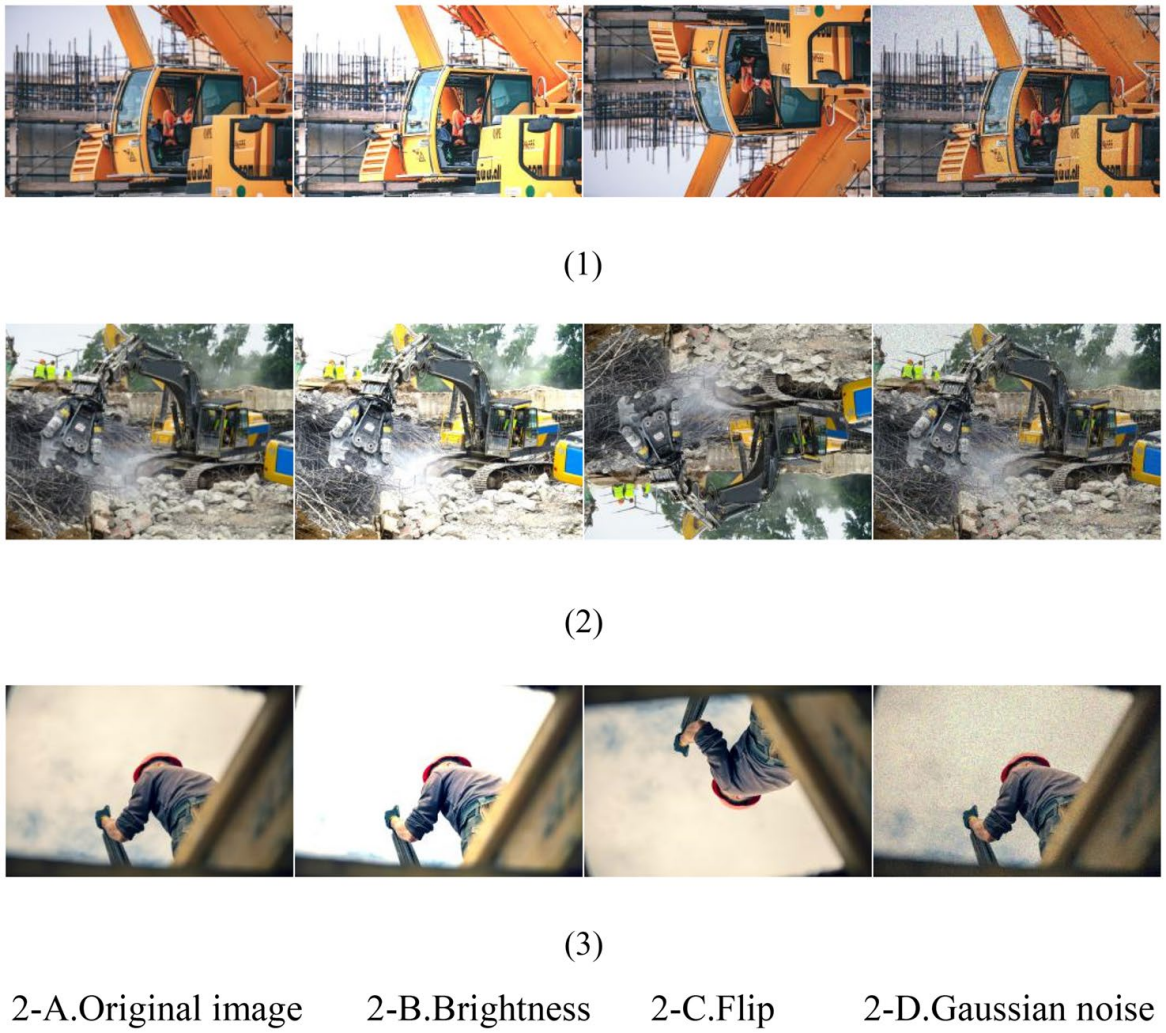
Data enhancement effect diagram.

**Figure 3:**
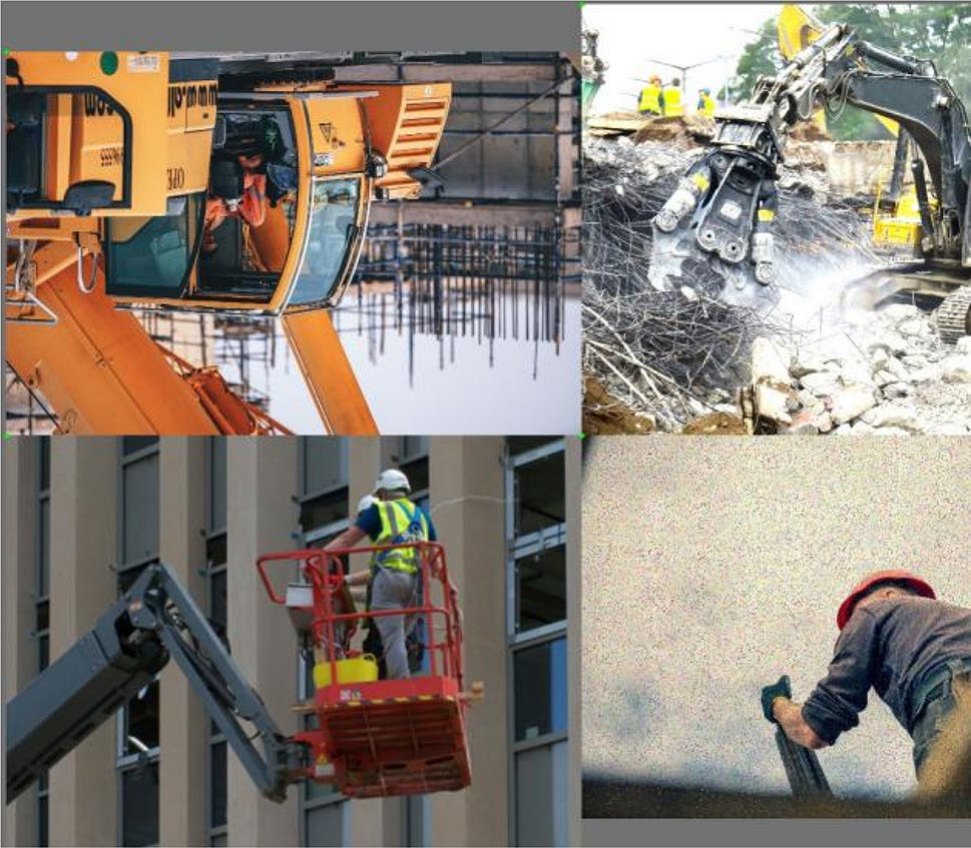
Mosaic data enhancement input example.

Data augmentation is a preprocessing technique used to broaden the image dataset. In this approach, the original image data is combined with the mosaic data augmentation method during input processing. Essentially, when multiple image datasets are stacked to create a new image dataset, they function similarly, with slight variations in operation. Mosaic data augmentation begins by randomly selecting four original image datasets, adjusting each image for brightness, zoom, flipping, noise addition, and other operations, as illustrated in Fig. 2. Subsequently, the four processed images are arranged, cropped, and zoomed freely, among other operations, and then pieced together to form a new image. An example of the input after Mosaic data augmentation is depicted in Fig. 3.

The Mosaic data augmentation method serves to expand the dataset's helmet feature data, significantly increasing the diversity of the detection dataset. This, in turn, enhances the detection algorithm's capability to identify small targets and improves its overall robustness. Moreover, this method allows for the simultaneous processing of data from four images, effectively expanding the dataset while minimizing computational overhead. As a result, it accelerates the detection speed and reduces the training time of the detection algorithm.

### B. Incorporating ECA channel attention mechanisms

The ECA attention mechanism is a variation of the attention mechanism used in image processing and computer vision tasks. It draws inspiration from the Squeeze-and-Excitation (SE) attention mechanism^30^, which enhances a model's feature representation by dynamically adjusting the significance of channels in CNN models.

The channel attention mechanism, applied to CNN models, aims to enhance a convolutional neural network's ability to capture relationships between channels. In traditional CNNs, the feature maps of each channel are typically considered as independent, with all channels assigned equal importance. However, in real-world images, different channels may exhibit varying relationships and levels of importance. The ECA attention mechanism addresses this by allowing the network to focus more effectively on task-relevant information through adaptive channel importance adjustments. This approach has proven highly effective in improving the model's capacity to represent critical channel features.

To be specific, the fundamental concept behind ECA channel attention involves assigning a weight to each channel. This is accomplished by introducing a learnable one-dimensional convolutional layer, which aggregates a global description of each channel by pooling the global average of its feature maps. For each input channel, a one-dimensional convolution operation is then applied using a convolution kernel, with the convolution result transformed into a weight value ranging from 0 to 1 via a sigmoid function to represent attention weights. Below is the ECA formula for generating channel weights through a one-dimensional convolution of size K:

1a $$ \omega = \sigma \left( {C1D_{K} \left( y \right)} \right) $$

where, C1D denotes one-dimensional convolution, *y* denotes channel,$\sigma$ denotes Sigmoid activation function.The larger the channel dimension,the larger the range of local cross-channel interaction. The mapping relationship between the channel dimension C and K is as follows:

1b $$ C = \phi \left( K \right) \approx \exp (\gamma \times K - b) $$

Given the channel dimension C, the kernel size K is determined adaptively:

1c $$ K \, = \Psi \left( C \right) = \left| {\frac{{\log_{2}^{(C)} }}{\gamma } + \frac{b}{\gamma }} \right|_{{{\text{odd}}}} $$

The parameters γ and b are hyperparameters in the context of the ECA attention mechanism. The γ parameter is employed to scale attention weights, allowing control over the concentration of attention distribution. A larger value of γ sharpens the attention, focusing it on a smaller number of channels, whereas a smaller γ broadens the attention distribution more evenly. Conversely, the b parameter is used to shift attention weights, influencing the reference point of attention. When b is a positive number, attention may be inclined towards channels with higher responses, while a negative b value may favor low-response channels. Typically, an initial value for b can be set, such as a smaller positive number like 1, and then adjusted based on experimental outcomes.

**Figure 4:**
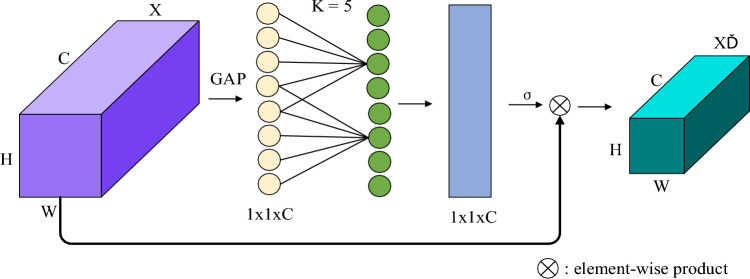
Schematic diagram of ECA module structure.

Finally, the features of each channel are multiplied by their respective attention weights to derive weighted channel features, as illustrated in Fig. 4. These weights are applied to each channel within the feature map, resulting in a weighted feature map where important channels are amplified, and less significant channels are suppressed. This approach enhances computational efficiency and boosts the performance of the ECA channel attention mechanism when compared to the SE channel attention mechanism.

With the incorporation of the ECA attention mechanism, the model becomes better equipped to adapt to relationships between different channels, address the correlation among channel features, and direct attention towards channels crucial for the task. This reduction in redundant information ultimately leads to improved model performance.

### C. Replacement of feature pyramid structure

Traditional Feature Pyramid Networks (FPNs) have certain limitations, including the loss of feature information and ambiguities. These limitations result in incomplete information transfer and a reduction in feature resolution. To address these issues, the Bidirectional Feature Pyramid Network (BiFPN)^14^ serves as an enhancement and extension of the conventional FPN.

The Weighted Bidirectional Feature Pyramid Network (BiFPN) is a multi-target, multi-size, and multi-scale target detection network structure. Rooted in efficient bi-directional cross-scale connectivity and weighted feature fusion, BiFPN constitutes a feature fusion network structure inspired by the theoretical foundations of PANet. This structure is re-engineered to facilitate feature fusion across the entire network, wherein features originating from top layers down to lower layers are fused. Instead of directly passing these features to the detector, the fusion process is conducted from the lower layers up to the top layers, with certain unfused features being pruned. In essence, it "redirects" the feature flow, allowing for enhanced feature fusion, cost savings, simplified network architecture, and rapid multi-scale feature integration.

BiFPN's design philosophy draws from the concepts of Feature Pyramid Network (FPN) and PANet. It facilitates the transmission of feature information between the upper and lower layers of the network. The network constructs a feature pyramid by employing both top-down and bottom-up pathways to merge features at varying scales. These top-down connections combine high-level features with their low-level counterparts, thereby extracting more comprehensive semantic information.

**Figure 5:**
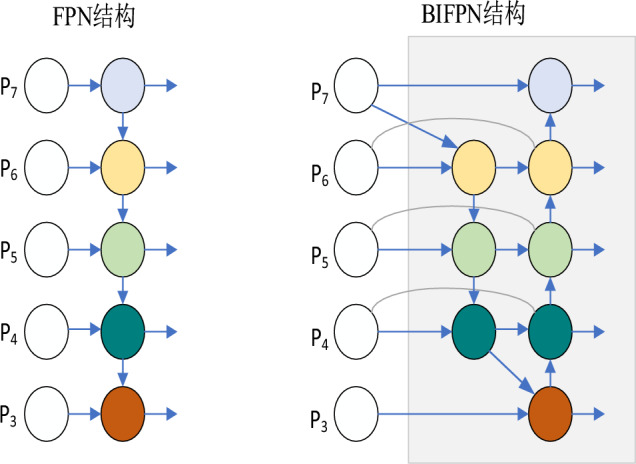
Structure of FPN and BIFPN.

The core concept behind BiFPN involves the gradual generation of high-level features layer by layer, starting from the initial bottom-level features. This process is achieved through bidirectional connectivity and layer-by-layer iteration. BiFPN introduces bidirectional connections, enabling feature fusion not only in top-down paths but also through the passage of features in bottom-up paths. This approach facilitates multilevel and multisize feature fusion and allows for the repetition of network layers. Consequently, it results in the acquisition of superior-quality semantic features, location features, and higher-level feature fusion, as illustrated in Fig. 5.

The computation of BiFPN feature fusion can be expressed as shown in Eq. 1d.

1d $$ {\text{F}}_{{{\text{out}}}} = \omega_{1} F_{1} + \omega_{2} F_{2} + \omega_{3} F_{3} + ... + \omega_{n} F_{n} $$

In the equation, F represents the layer feature, ω denotes the feature weight for the layer, and Fout represents the resulting output feature following feature fusion.

Furthermore, as a feature fusion network, BiFPN addresses the issue of varying importance levels among connected features at each scale and layer in the final output features. It achieves this by incorporating weight parameters designed to learn and update the contributions of different input features, thus differentiating their degrees of importance. Simultaneously, the overall network structure shares these learned weight parameters. This approach allows us to balance the information from features at different scales, resulting in higher-quality fused features for subsequent detection stages.

This bidirectional approach proves highly effective in capturing feature information at different scales, enabling the construction of feature pyramids at various scales. This solution ensures a more stable and consistent detection performance across different levels, making it a common feature in advanced target detection models such as EfficientDet and RetinaNet. Models like BIFPN that utilize this approach have consistently delivered outstanding performance and results.

### D. YOLOX decoupling header

In the realm of target detection, the classification task and the regression problem are a pair of conflicting objectives. This is primarily because the characteristics required for classification differ from those needed for regression. Classification necessitates a degree of subtlety, while regression relies more on contour and boundary features.

The concept of a shared head structure originally emerged in the Fast RCNN paper, offering a one-step solution that significantly enhances detection speed. However, as single-stage and two-stage detection networks evolved, some classical target algorithms continued to employ coupled detection heads. Researchers gradually realized that this coupled structure could potentially compromise performance. It was observed that during backpropagation, coupling could lead to slow network convergence and reduced accuracy. Consequently, researchers began exploring alternative approaches to enhance both detection speed and effectiveness.

As the applications of the YOLO series have expanded, particularly with the evolution of the backbone and feature pyramid (e.g., FPNPAN), the concept of a decoupled head structure has been introduced. This concept aims to strike a balance between detection effectiveness and speed while avoiding a substantial increase in computational demands. It recognizes that classification and localization pertain to distinct aspects of the task.

**Figure 6:**
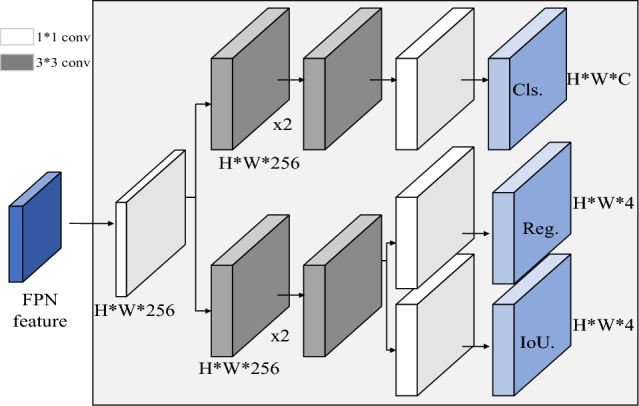
YOLOX decoupling header.

One notable implementation of the Decoupled Head structure is found in YOLOX^31^, as depicted in Fig. 6. From a foundational perspective, it begins with a 1 × 1 dimensionality reduction operation, followed by the connection of two branches: one for classification and the other for localization. This approach separates the extraction of target location and category information, allowing them to be learned independently within different network branches. Subsequently, these branches are fused, effectively reducing the number of parameters and computational complexity. This enhances the model's generalization ability and robustness.

By incorporating the YOLOX decoupled head into YOLOv5, we not only integrate the strengths of both algorithms but also elevate detection performance and accuracy.

In real-world datasets and task requirements, fine-tuning operations such as hyperparameter tuning, data augmentation, and the use of appropriate loss functions and optimizers across the entire network become essential. We have observed that substituting the YOLOX head with a decoupled head significantly improves convergence speed and enhances model performance, leading to improved detection accuracy. Consequently, decoupled classification and regression heads are widely adopted in most primary and secondary detectors, particularly in multi-class target detection scenarios, resulting in superior detection performance.

### E. Evaluation indicators

For target detection algorithm models, algorithms are usually evaluated for performance using some evaluation metrics, such as Precision (Pr), Recall (Rc), F1 Evaluation Score (F1), Average Precision (AP), Mean Average Precision (AP), Mean Average Precision (mAP), and Frames Per Second (FPS)^32^ , among others.

Precision and recall are a pair of metrics used in machine learning to measure how accurate a classifier is. Precision is also known as positive predictive value and Pr is called positive predictive value. It represents the proportion of the number of correct predictions when the predicted detection frame overlaps with the real detection frame. Similarly, Rc represents the proportion of the number of correct predictions across all real objects.The curves of Pr and Rc emphasize the trade-off between precision and recall by giving a comprehensive view. When a comprehensive view of Pr and Rc is required, then F1 is proposed.F1 is a combined measure of precision and recall. It considers their average values to represent the difference in detection performance. And FPS is a metric of detection speed, which indicates the number of images that can be processed per second by the network of object detection algorithms. Pr, Rc, and F1 are calculated as expressed in Eqs. (2a), (2b), and (2c).

2a $$ Pr \, = \, \frac{TP}{{TP + FP}} $$

2b $$ Rc \, = \, \frac{TP}{{TP + FN}} $$

2c $$ F1 \, = \, \frac{2 \times Pr \times Rc}{{Pr + Rc}} $$

In the above equation, TP denotes True Positive, which represents correct detection of an object; FP denotes False Positive, which represents detection of an incorrect object as a correct object; and FN denotes False Negative, which represents detection of a correct object as an incorrect object.

The value of average precision ranges from 0 to 1. A threshold value is set for AP before starting the calculation, and then the IoU of the detection results is calculated and sorted. The detection results that are categorized correctly and at the same time the IoU reaches the set value are grouped into TP, and the FP and FN are calculated in the same way, and then the Pr and Rc of this category under this set condition are calculated. adjusting the set threshold, the same operation is repeated to get the Pr and Rc under each set threshold, and the Pr-Rc (PR) curve is drawn, and the size of the area underneath the PR curve is the AP, while the The average value of the AP of all the detected objects in the target detection application scenario is the mAP.The formulas for calculating the AP and mAP are shown in Eqs. (2d) and (2e).

2d $$ AP = \int_{0}^{1} {Pr} (Rc)dRc $$

2e $$ mAP = \frac{{\sum\limits_{i = 1}^{n} A P_{i} }}{n} $$

Evaluation metrics serve as critical indicators for assessing and validating the performance of an object detection algorithm. A top-tier object detection algorithm should not only yield high mean Average Precision (mAP) and F1 scores but also excel in various other evaluation metrics.

In this paper, we aim to enhance the YOLOv5 object detection algorithm network framework by integrating BiFPN, a weighted bidirectional feature pyramid network. This integration facilitates the transmission of both semantic and positional information within the network's feature fusion structure. Consequently, the lower-level prediction output branch gains access to a more comprehensive semantic feature graph, while the top-level prediction output branch benefits from a richer set of front and back logical information within the feature graph. This augmentation contributes to an overall improvement in the algorithm network's detection accuracy.

## Experiment and results

### A. Experimental environment

All experiments were conducted on a Windows operating system using an NVIDIA GeForce RTX 3090 GPU equipped with 16 GB of video memory. The YOLOv5 model was implemented within the PyTorch deep learning framework, utilizing Python 3.8 as the programming language. PyTorch version 1.10.0 was employed, alongside CUDA 11.1.1 for GPU acceleration.

For the experiments, we utilized an open-source and in-house helmet dataset comprising a total of 23,088 images. This dataset was partitioned into training and test sets in an 8:2 ratio. The images were annotated in the VOC format and subsequently transformed into YOLOv5-compatible TXT format. The dataset labels encompass two categories: individuals wearing helmets, designated as "Helmet," and those not wearing helmets, labeled as "Head."

Table 1 outlines the hyperparameters essential for the experiments.

**Table 1:** Parameter setting.

Parameter name	Parameter value
Momentum	0.937
Weight decay	0.0005
Batch size	16
Learning_rate	0.01
Epochs (number of iteration rounds)	300

### B. Training visualization

**Figure 7:**
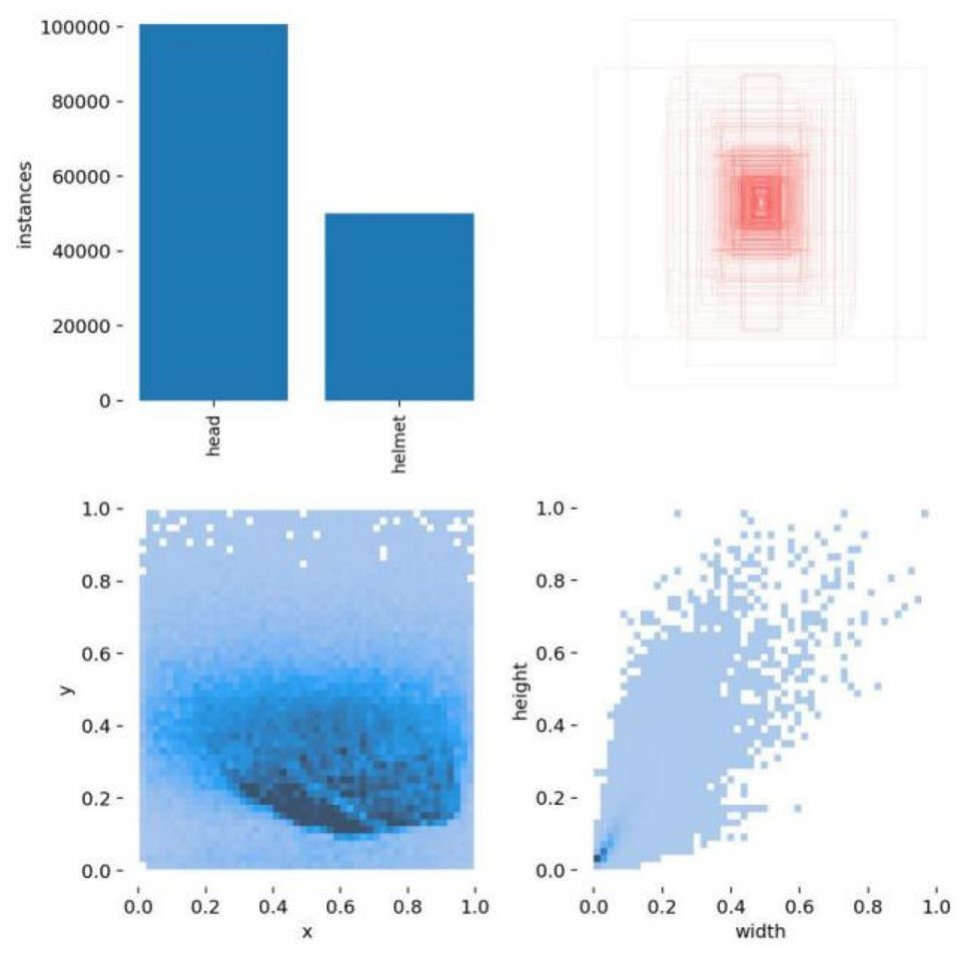
Distribution of label visualization.

The dataset label visualization distribution graph is a valuable tool for depicting the distribution of samples across various categories within the dataset, as exemplified in Fig. 7 below. Positioned in the upper left corner are the dataset's object categories, providing a comprehensive overview of the data and highlighting the balance of samples among these categories. In the bottom left corner, the distribution of object centroid locations is depicted, emphasizing the focal points and location information of the samples.

Moving to the top right plot, it showcases the positions of horizontal and vertical coordinate centroids, aiding in the detection of any dataset skewness. Lastly, the bottom right plot illustrates the distribution of object sizes, where the horizontal coordinate WIDTH and the vertical coordinate HEIGHT represent the width and height of the objects. This offers valuable insights into assessing the dataset's usability and reliability.

Visualizing the model training process allows us to monitor the model's performance metrics in real time. By plotting these metrics as the number of training iterations increases, we gain insights into the model's learning progression, including metrics like accuracy and loss functions. This monitoring helps in preventing overfitting and aids in devising an effective tuning strategy.

Furthermore, visualizing the training process enables us to comprehend parameter and gradient variations within the model, optimizing learning rates and weight initialization strategies. This process also evaluates the effectiveness of techniques such as data augmentation and regularization. By comparing augmented data samples with their original counterparts, we can assess the impact of augmentation on the dataset's diversity and the model's robustness.

**Figure 8:**
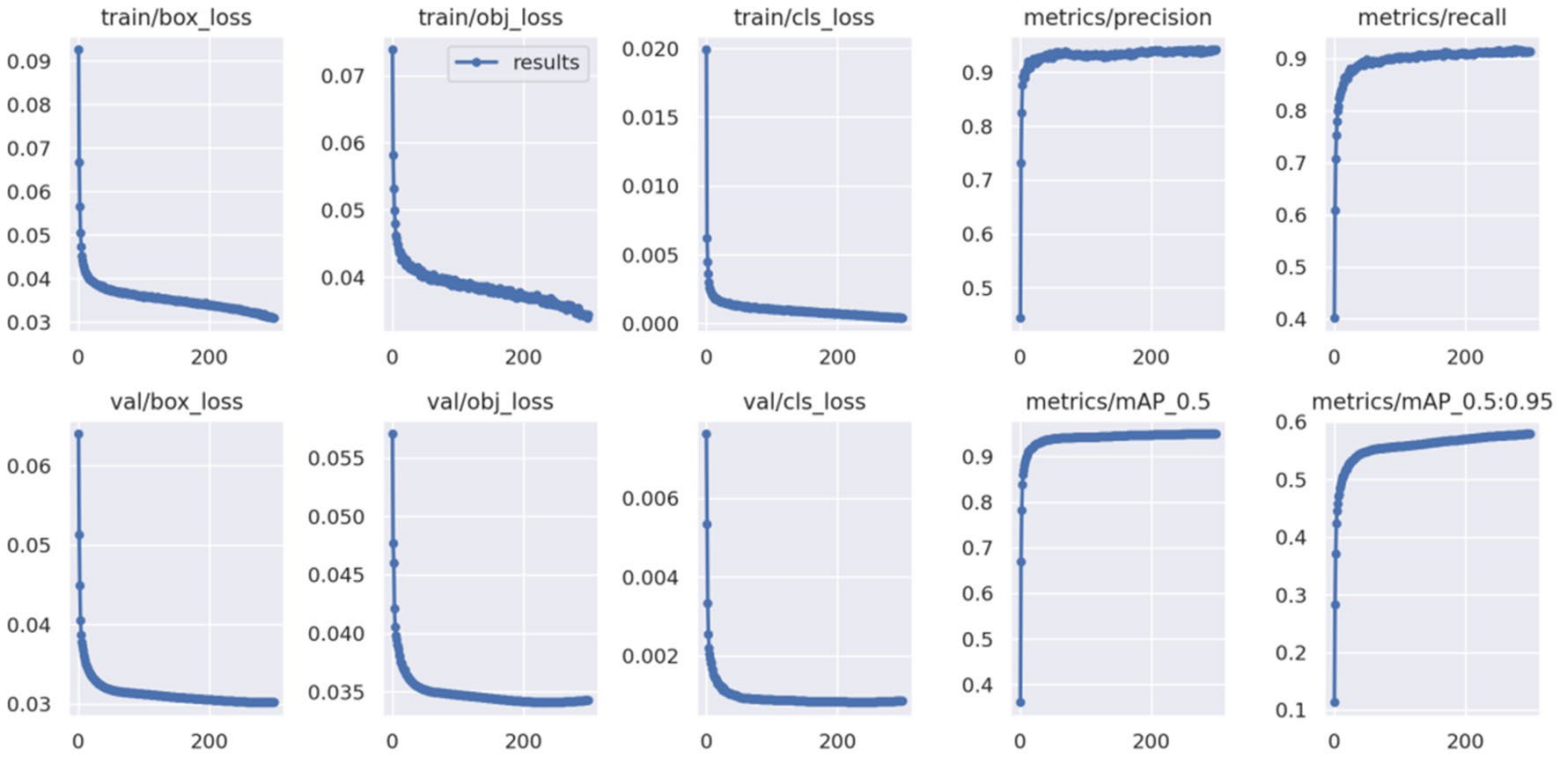
Improved visualization of the model training process.

Figure 8 showcases the visualization of the improved YOLOv5 algorithm's performance over 300 rounds of training on the dataset. Notably, the precision and recall metrics exhibit minimal fluctuations. The loss curves for both the training and validation sets converge gradually without significant fluctuations, encompassing bounding-box regression loss (box_loss), objective confidence loss (obj_loss), and classification loss (cls_loss). These trends indicate that the model neither overfits nor underfits during training. The values of detection precision metrics, including mAP_0.5 and mAP_0.5:0.95, steadily rise and stabilize, underscoring the optimized model's robust learning capabilities.

### C. Experimental results

**Figure 9:**
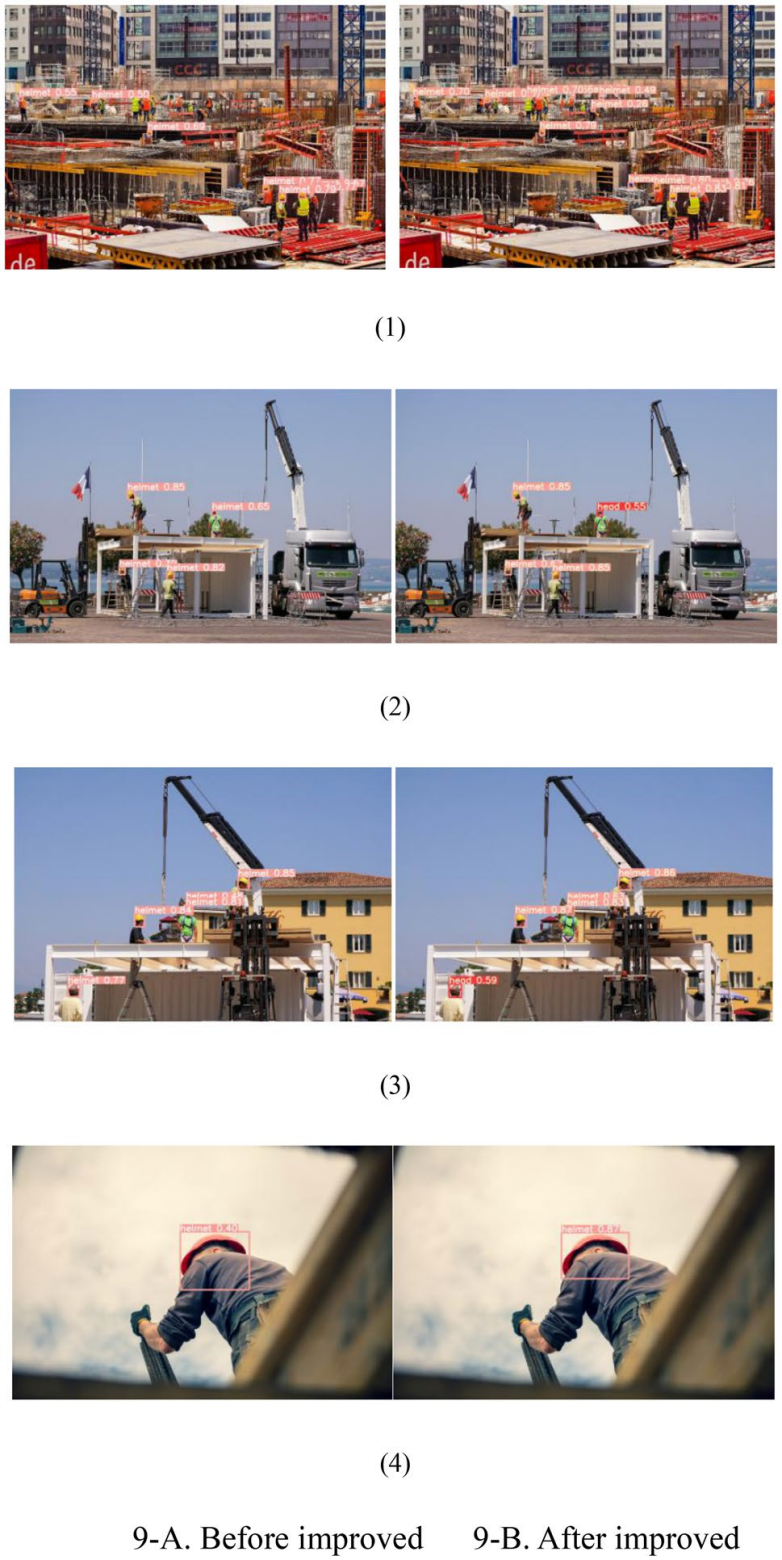
Comparison of the detection effect of the improved algorithm.

1. The following is a comparison of the results of BiFPN feature fusion structure, as shown in Table 2
2. The algorithm comparison results of the improved model are shown in Table 3
3. Improve the image comparison results, as shown in Fig. 9
4. Analysis of results

**Table 2:** The results in comparative experiments of BiFPN feature fusion structure.

Arithmetic	mAP %
YOLOv5	92.9
YOLOv5-BiFPN	95

**Table 3:** Experimental results of algorithm comparison for improved models.

Model	Weight (MB)	mAp (%)	Recall (%)	Precision (%)
Yolov5s	13.6	92.9	94.0	94.5
Yolov5s + ECA	14.0	92.2	88.4	94.1
Yolov5s + SE	13.7	95.0	91.6	94.4
Yolov5x	87.1	95.7	93.1	95.0
Improved Yolov5	27.9	95.9	92.4	94.7

Based on the results of the comparative experiments, it is evident that the selected improved BiFPN network structure exhibits a notable 2.1% increase in mAP value compared to the original YOLOv5s algorithm. The detection performance of the improved algorithm, YOLOv5-BIFPN-ECA-HEAD (YOLOv5-BEH), surpasses that of the original YOLOv5s algorithm, with a remarkable 3.0% rise in mAP value. Although the change in detection performance of the improved model may not be as pronounced when compared to the network structure of the original YOLOv5x algorithm, this thesis contends that the concept of cross-layer connectivity and weight control within BiFPN serves as a valuable guideline for enhancing the detection performance of target detection algorithms. Additionally, it offers a lighter deployment weight than YOLOv5x.

Consequently, this thesis leverages the BiFPN feature fusion structure to bolster the algorithm's feature fusion capability by regulating the weight ratio. This is achieved by connecting to various layers within this network structure through channel dimension splicing, subsequently fusing features based on their varying degrees of importance, thus capturing more high-level information. Moreover, comparative assessments of various attention mechanisms reveal that selecting YOLOv5-BEH as the improved model results in the model's ability to focus on small targets, leading to higher average precision and enhanced accuracy while maintaining a commendable recall rate. Additionally, the adoption of a decoupling head serves to expedite the model's convergence speed and further elevate the algorithm's detection performance.

As illustrated in Fig. 9, a comparative analysis of the improved algorithm's detection effectiveness clearly demonstrates that the YOLOv5-BEH model outperforms the YOLOv5 model across various performance aspects, particularly in intricate and diverse detection scenarios. In scenarios involving small and densely-packed targets, where the YOLOv5 model is prone to omitting and misdetecting, the YOLOv5-BEH model excels. It exhibits higher target localization accuracy and robustness within complex construction scenarios. Consequently, the YOLOv5-BEH model exhibits superior performance and enhanced target localization accuracy.

## Conclusion

In this paper, we introduce an enhanced algorithm model, YOLOv5-BEH, designed to address the limitations observed in current helmet detection algorithms. These limitations encompass challenges related to false detections, especially leakage misdetections, and low accuracy, particularly in scenarios involving small and densely-packed targets.

Our improved algorithm replaces the BiFPN feature pyramid structure within the YOLOv5 backbone extraction network for feature fusion. We introduce the ECA channel attention mechanism and refine the loss function to incorporate the decoupling header, resulting in enhanced detection speed and the accurate identification of small or densely-packed targets. Consequently, this significantly improves the model's generalization ability and robustness. The experimental results indicate that the YOLOv5-BEH model effectively mitigates false and leakage detections, achieving high-accuracy detection that meets the stringent requirements for helmet-wearing detection in complex construction scenarios when compared to the YOLOv5 model. Moreover, the model demonstrates robust generalization, making it applicable to various target detection tasks.

In our ongoing work, we plan to further optimize the model and deploy it in real-world projects. We aim to gradually extend its application to diverse areas, effectively addressing complexities and variations encountered in detection tasks, such as varying lighting conditions, crowd sizes, target distances, and more. This approach will enable us to achieve faster and more accurate detection results in a wide range of scenarios.

## Data Availability

The first statement confirms that all methods have been carried out in accordance with the relevant guidelines and regulations, that all experimental protocols have been approved by the designated Guangdong Polytechnic Normal University, School of Electronics and Information Technology, and that informed consent was confirmed to have been obtained from the team and participating members. As the dataset supporting the results of this study is one of the core competencies of the college team, the availability of these data is restricted. These data were used under licence for this study and are therefore not publicly available, but may be obtained from the corresponding authors upon reasonable request and with the permission of the team for academic research purposes only. In addition, the University has a Graduate School as the Ethics Committee and the President's Office as the Institutional Review Board, and the full name of the Institutional Review Board that approved the research is the Office of the President of the Guangdong Polytechnic Normal University with a clear statement that it covers all experimental methods.
